# The Value of Human Life

**Published:** 1911-03

**Authors:** 


### The Value of Human Life

THE antivivisectionists and the antivaccinationists, always with us and always crying out for mercy to the poor dumb animals who are sacrificed to the cruel brutalised instincts of the medical profession, have been given substantial food for thought recently in two widely separated directions.

In England, Lord Cromer, and there is no more judicious or careful authority, has made public some illuminating statistics. In an Indian district with a population of 827,000 four months prior to the appearance of bubonic plague 187,000 people were inoculated against the disease leaving 640,000 unprotected. When the plague came 314 deaths occurred among those inoculated. Among the unprotected there were 29,723 deaths. Reduced to understandable figures only one person in every 595 of those protected died; of the others who did not avail themselves of the advance in scientific prevention one in every 22 died.

The other instance comes from Dr. W. C. Gorgas of the United States army, in charge of the canal zone who states that since 1905 there have been no cases of yellow fever or bubonic plague on the isthmus and that malaria among the canal employes has been reduced from 821 per thousand to 215.

In spite of these patent facts the faddists and purblind objectors to the protection of humanity continue to careen through otherwise sensible and interesting journals and magazines and give forth page on page of literary hysteria to the crusade for the protection of the "poor dumb brute" against the brutalising influences of medical research.

It were useless waste of space to call attention to the malicious fabrications of some of the descriptions of horrible scenes which these over enthusiastic laborers in self chosen charities claim to have seen; they are too well known; but it was a clever damper to the enthusiasm of these somewhat human crusaders which an insistent layman spread over their exuberant if misguided efforts when he asked: "At how many rabbits or guinea pigs do you value your wife or your husband or your child?"

